# The Relationship between Bullying and Emotional State among Undergraduate Nursing Students: A Cross-Sectional Correlation Study

**DOI:** 10.1155/2023/2397229

**Published:** 2023-08-05

**Authors:** Nada A. AbuAlula, Abdulaziz Mofdy Almarwani, Daniel Mon Mamanao, Naif Salem Altarawneh, Mohammed R. Alharbi, Inas A. Ebeid

**Affiliations:** ^1^Department of Psychiatric Nursing, College of Nursing, Taibah University, Medina, Saudi Arabia; ^2^Psychiatric Nursing and Mental Health Department, Port Said University, Port Fuad, Egypt

## Abstract

**Introduction:**

Bullying behavior by nursing students is a serious problem that has contributed to the drop in numbers in the nursing profession.

**Aim:**

The study investigated the relationship between bullying and the emotional state of undergraduate nursing students.

**Methods:**

The study used a cross-sectional correlation design with a sample of 286 undergraduate nursing students from multiple nursing colleges located in the western region of Saudi Arabia. The 21-item depression, anxiety, and stress scale (DASS-21) was used to measure the study outcomes. A revised version of the Bullying Assessment Questionnaire was used to assess bullying experiences. Statistical analyses, including a *t*-test, Pearson correlation coefficient, and a one-way between-subjects ANOVA, determined the significance of the relationship between study variables.

**Results:**

There was a high prevalence of depression, anxiety, and stress symptoms among Saudi nursing students. Most of the respondents reported mild to extremely severe symptoms of depression (58.7%), anxiety (58%), and stress (44.8%). Around 90.1% of the nursing students reported exposure to a form of bullying over the previous 12 months. The most common items reported as part of this behavior included continually being assigned tasks beyond their capacity. Family members were the most frequently reported source of bullying (29.8%), followed by nursing faculties (20.9%). Bullying behavior was positively correlated with students' scores for depression, *r* (284) = 0.49, *n* = 286, *p* < 0.01; anxiety, *r* (284) = 0.54, *p* < 0.01; and stress, *r* (284) = 0.56, *p* < 0.01.

**Conclusion:**

The study's findings raise concerns and highlight the importance of decreasing the risk of depression, anxiety, and stress among undergraduate nursing students. Nurse educators must ensure that students receive psychological support to decrease these psychological outcomes. Regularly monitoring bullying behavior is essential to maintain students' psychological stability, which could eventually reduce professional dropout rates.

## 1. Introduction

Bullying is intentional and persistent violence by a perpetrator towards a peer. It may involve exploiting a physical, psychological, or social power imbalance, and a student may become a habitual victim [1]. It is a severe problem affecting nursing students and has contributed to the falling number of individuals in the nursing profession [1]. Bullying is a serious problem in nursing education, both in person and online. In nursing colleges, many cases are underreported because most students believe it is a normal part of their nursing education and are unaware of the school policies to address this issue [2]. Year-on-year, there is an increasing rate of bullying of online users in the form of videos, images, and words that may threaten, mock, or insult others. This often occurs among students who use social networking sites, chat rooms, and instant messaging applications. Students are often unaware that using social media can lead to cyberbullying [1, 3].

Bullying has short-term and long-term negative impacts on a student's well-being, academic performance, social life, and mental health [4, 5]. The negative impacts of bullying include emotional stress, dissatisfaction, fewer interests, self-doubt, decreased commitment, and confusion. These often lead to students believing that higher education institutions offer little or no support in solving the problem [4], causing high levels of anxiety and depression [6]. In a study by Ahmed et al. [7], females had 2.9 times more negative emotional reactions to bullying than males. Victims with social support experienced lower levels of emotional stress. In addition, poor quality of life significantly contributed to bullies' mindsets. Compliance with the academic requirements and hospital duties combined with the stress of studying for classroom examinations add further stress that may reduce students' tolerance to unpleasant environments [5].

Bullying covers four possible dimensions, including physical bullying (e.g., kicking, hitting, pushing, grabbing, and destroying objects), verbal bullying (e.g., name-calling, teasing, abusing, and mocking), relationship bullying (e.g., excluding from social situations and spreading rumours), and cyberbullying [8]. A study conducted by Bambi et al. (2018) revealed that bullying is a predictive factor for burnout (*β* = 0.37 *p* < 0.001) and shows a negative correlation with job efficiency (*r* = −0.322, *p* < 0.01). Victims are twice increased in comparison to nonvictims (95% CI: 1.3–1.7). 78.5% of bullied nurses with 5 years less of service leave their jobs [9].

### 1.1. Bullying in the Nursing Field

Nurses are exposed to bullying through many channels, including their patients, patients' relatives, classmates, doctors, and other medical staff. Some students view themselves as participants who cause student-faculty bullying [10]. Bullying can be a self-perpetuating cycle in the nursing profession. students who are bullied often go on to bully a colleague they believe to be powerless. This leads to the pervasiveness of bullying in nursing culture [11]. Studies have revealed that the most notorious source of bullying is not students' classmates but faculty members [12–14].

Although the nature of bullying differs across contexts, it undoubtedly occurs in the nursing profession. In the clinical setting, nurses work in a toxic environment and perceive students as added workload [15]. Student nurses encounter this behavior, often have inadequate knowledge, and feel pressured by the new environment [16]. Existing literature has mainly explored bullying among students in clinical training; only a small number of studies have investigated bullying in nursing students outside of this setting [2, 17–21]. However, bullying has become a global concern, affecting more than 60 million workers in the United States. There has been a lack of information about the impact of academic harassment on conflict management in higher educational institutions [1].

A review on the impact of transition programs (TPs) on workplace bullying, violence, stress, and resilience for students and new graduate nurses (NGN) reveals that there is a need to improve nursing student's transition to clinical practice plus their resilience to overcome bullying [22]. Among the total of 779 studies, after rigorous filtering, 19 were found to fit the inclusion criteria. Most are female (80%–100%), and their ages ranged between 21 and 54 years old predominantly Associate Degree in Nursing (ADN) and Bachelor of Science in Nursing (BSN) Degree. The stress score for NGNs who participated in the TP approach had a significant difference (*p* = 0.050); 90% of the NGNs (*n* = 61) reported that resilience sessions within the TPs were helpful and that their stress scores decreased [22].

Studies on nursing students' bullying-related experiences in Saudi Arabia have been limited [23–25]. According to Ullah et al. [26], surveys should be conducted in Saudi Arabia to provide insights into the educational experience. This could help identify bullying at an early stage. Therefore, this study aimed to investigate the relationship between bullying and the emotional state of undergraduate nursing students at universities in the western region of Saudi Arabia. The study also seeks to identify sources of bullying among nursing students and students' actions towards bullying. The findings will increase awareness of bullying among administrators, educators, clinical leaders, and instructors.

This research seeks to answer the following questions:

1. How frequently do nursing students experience bullying behavior?
2. How frequently do nursing students experience depression, anxiety, and stress symptoms?
3. Is there a significant relationship between bullying and nursing students' anxiety, depression, and stress?
4. Is there a significant difference between year levels in the nurses' characteristics, the bullying behavior they have experienced, and their emotional state?
5. What are the predictors of students' anxiety, depression, and stress?
6. What are the sources of bullying and actions taken by nursing students towards bullying?

## 2. Methodology

### 2.1. Study Design

The study used a cross-sectional correlational design to facilitate data collection from the target population at a single point in time without influencing the collected variables [27].

### 2.2. Sample

This study sampled 286 students enrolled in undergraduate nursing programs in universities in the western region of Saudi Arabia. An electronic invitation was sent to all students in these nursing colleges. All eligible participants who completed the questionnaire were included in the study. The study inclusion criteria were full-time undergraduate nursing students enrolled in the 2020/2021 academic year. The sample size was calculated using G-Power. The parameters used to estimate the sample size included effect size (*f*2) = 0.20; alpha = 0.05; power = 0.95. The total sample size based on this approach was 262 participants.

### 2.3. Data Collection Tool

The researchers used a self-reported electronic version questionnaire as a data collection tool. The questionnaire comprised four parts. The first part covered the respondents' demographic data, including their gender, nationality, age, and marital status. The second part used the Bullying Assessment Questionnaire. The researchers developed this tool after a thorough literature review [24, 28]. It is a 30-item questionnaire that assesses the bullying experiences of nursing students. Five possible responses (never, seldom, sometimes, frequently, and always) are represented by scores 0, 1, 2, 3, and 4. The subcategories were computed by summing the items to calculate the total score. The total possible score was 120. To establish face validity, the researchers asked five experts in nursing education and psychiatric nursing to ensure the statements' clarity and relevance to the questionnaire's goal. The researchers then revised the questionnaire. They conducted a pilot study with 30 students to examine the questionnaire's reliability, which had a Cronbach alpha of 0.88.

The third part used the Arabic version of the 21-item depression, anxiety, and stress scale (DASS-21), a self-reported measure of DAS experience based on a dimensional measurement of psychological disorders [29]. The DASS-21 consists of four possible responses (never, sometimes, often, and almost always) represented by scores of 0, 1, 2, and 3. It includes 7 items for each category. According to Coker et al. [30], the DASS-21 has excellent reliability and produces Cronbach's alpha values of 0.81, 0.89, and 0.78 for the categories of depression, anxiety, and stress, respectively [28]. Summing the items computes the categories and calculates a total score. The researchers obtained a letter of permission from the original author. The fourth part was an open-ended questionnaire about how students perceived the consequences of bullying in their everyday life.

### 2.4. Data Collection

The researchers distributed the questionnaire electronically to the participating nursing students. When they agreed to participate, the site immediately opened the questionnaire page. Data collection took two months, and the researchers followed up on the nurses' responses as necessary. The questionnaire was distributed via e-mail and social media (WhatsApp). There was no time limit for students to complete the questionnaire. However, during the pilot testing of the questionnaire, it was clear that a minimum of 15 minutes was sufficient to complete the questions.

### 2.5. Data Analysis

The researchers coded and entered the data using the Statistical Package for Sciences (SPSS 25). They used descriptive statistics, including the mean, standard deviation, and frequency, to analyze the demographic data. Statistical analyses, including a *t*-test, Pearson correlation coefficient, multipl linear regression, and a one-way between-subjects ANOVA, determined the significance of the relationship between study variables.

## 3. Results

### 3.1. Demographic Characteristics

The study involved 286 nursing students who agreed to participate and completed the online questionnaire (Table 1). The mean age of the nursing students was 21, and the majority were female (82.5%), single (95.8%), and from Saudi Arabia (99%). Nearly half of the nursing students (43%) were in their fourth academic year, and 32.9% were in their third academic year. Almost half of the nursing students reported not reading the student rights manual (46.5%). Most nursing students (57%) stated that bullying did not negatively affect their appreciation of the nursing profession. However, 19.2% responded with yes, and 23.8% responded with unsure.

### 3.2. Prevalence of Bullying among Nursing Students

Nursing students' bullying experiences were assessed using the 30-item bullying questionnaire, adapted, and modified based on previous research. The 30-item bullying questionnaire showed excellent reliability with a Cronbach alpha of 0.95. The highest possible score for the 30-item bullying questionnaire was 120. The prevalence of bullying reported in the sample was relatively low (*M* = 22.9, SD = 19.6).  Table 2 describes the frequency of bullying behaviors that nursing students reported for each of the 30 items on the questionnaire. The most common bullying behaviors that students reported included experiences of continually being assigned tasks beyond their capacity (*M* = 1.44, SD = 1.40), receiving unfair evaluations of their work (*M* = 1.40, SD = 1.20), and other people underestimating the value of their academic work (*M* = 1.22, SD = 1.18). The least common bullying items that nursing students reported were being physically assaulted (*M* = 0.11, SD = 0.39), being treated badly or unfairly because of their race (*M* = 0.67, SD = 0.23), and being treated badly or unfairly because of incapacities or weaknesses (*M* = 0.72, SD = 0.34).

### 3.3. Prevalence of Depression, Anxiety, and Stress among Nursing Students

The prevalence of depression, anxiety, and stress among nursing students was measured using the DASS-21 (Table 3). The average total DASS-21 score in the sample was *M* = 20.9 (SD = 15.7) out of possible 84. In addition, the average scores on the three subscales of depression (*M* = 7.34, SD = 5.91), anxiety (*M* = 5.94, SD = 5.19), and stress (*M* = 7.62, SD = 5.67) were within the normal range. However, almost one-third of study participants reported abnormal scores on all three subscales, as shown in Table 3.

### 3.4. The Relationship between Bullying Behavior and DASS-21

The Pearson correlation coefficient was used to assess the relationship between bullying behavior and DASS-21 scores (Table 4). The results showed a significant positive association between bullying behavior and DASS-21 scores, *r* (284) = 0.57, *p* < 0.01. Bullying behavior was also positively correlated with students' scores for depression, *r* (284) = 0.49, *n* = 286, *p* < 0.01; anxiety, *r* (284) = 0.54, *p* < 0.01; and stress, *r* (284) = 0.56, *p* < 0.01.

Multiple regression analysis examined the relationship between students' DASS-21, bullying behavior, and other demographic variables (gender, age, marital status, and current academic year). Specifically, the analysis examined whether bullying behavior and other demographic variables can predict students' stress using the DASS-21 score. The model was significant *F* (5, 280) = 24.91, *p* < 0.001. The model explained 30% (*R*^2^ = 0.301) of variance in the outcome variables. Students bullying behavior (*B* = 0.13, *t* = 10.9, *p* < 0.001), age (*B* = −0.30, *t* = −1.99, *p* = 0.04), and marital status (*B* = 2.56, *t* = 2.14, *p* = 0.03) contributed significantly to the model.

Furthermore, the multiple regression analysis examined whether bullying behavior and other demographic variables can predict students' anxiety using the DASS-21 score. The model was significant (*F* (5, 280) = 22.92, *p* < 0.001) and explained 29% (*R*^2^ = 0.290) of variance in the outcome variables. Students bullying behavior (*B* = 0.13, *t* = 10.3, *p* < 0.001), age (*B* = −0.33, *t* = −2.08, *p* = 0.04), and marital status (*B* = 2.71, *t* = 2.08, *p* = 0.03) contributed significantly to the model.

Finally, multiple regression analysis examined whether bullying behavior and other demographic variables can predict students' depression using their DASS-21 scores. The model was significant (*F* (5,280) = 21.0, *p* < 0.001). The model explained 27% (*R*^2^ = 0.273) of variance in the outcome variables. Students bullying behavior (*B* = 0.15, *t* = 9.68, *p* < 0.001), age (*B* = −0.50, *t* = −1.77, *p* = 0.008), and marital status (*B* = 3.33, *t* = 2.23, *p* = 0.02) contributed significantly to the model.

### 3.5. Nursing Students' Academic Year and Bullying Behavior

A one-way between-subjects ANOVA was conducted on bullying behavior and DASS-21 scores across students in four academic years (first, second, third, and fourth years) (Table 5). The results showed that bullying significantly impacted students' total DASS-21 scores (*F* (3, 282) = 5.38, *p* = 0.014). Post-hoc comparisons indicated that the mean score for bullying behaviors in students enrolled in their third academic year (*M* = 27.57, SD = 19.6) was significantly different compared to students in other academic years (*p* = 0.014). Furthermore, students' total DASS-21 scores were statistically significant across the academic years (*F* (3, 282) = 4.71, *p* = 0.003). Post-hoc results showed that students in the third academic year reported higher DASS-21 scores (*M* = 25.57, SD = 14.5). Differences in students' depression (*p* = 0.001), anxiety (*p* = 0.031), and stress (*p* = 0.003) scores were statistically significant across the four academic years. Specifically, third academic year students' scores for depression (*M* = 9.06, SD = 5.60), anxiety (*M* = 7.12, SD = 5.01), and stress (*M* = 7.89, SD = 4.53) were higher compared with students in other academic years (Table 5).

### 3.6. Sources of Bullying and Action Taken by Students

**Figure 1:**
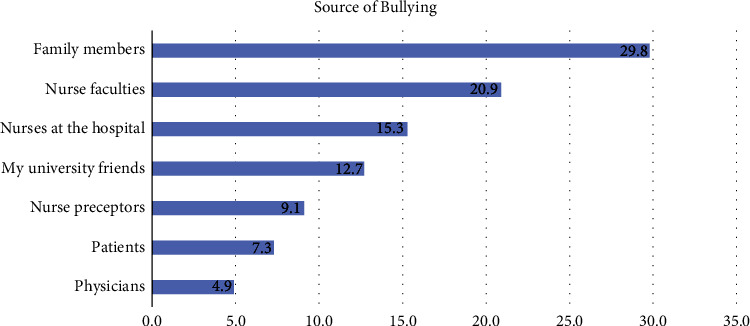
Student identified source of bullying.

Nursing students were asked about the source of bullying they had experienced (Figure 1). Most students indicated that family members were the most significant source of bullying (29.8%), followed by nursing faculty members (20.9%), nurses at the hospital (15.3%), and university friends (12.7%).

**Figure 2:**
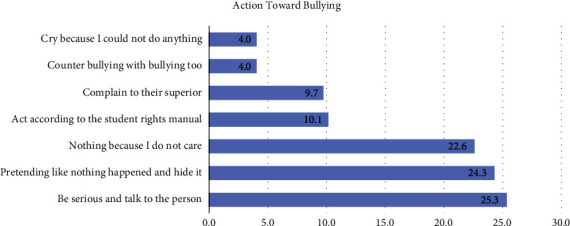
Students' action towards bullying.

Students were also asked about their actions towards bullying (Figure 2). One-quarter of nursing students reported that they pretend not to be upset and hide their discomfort when facing bullying (25.3%). Around 24.3% of students said they become more serious and talk to the person bullying them (24.3%). Other students indicated that they do not care about bullying (22.6%), act according to the student rights manual (10.1%), or complain to their superior (9.7%)

## 4. Discussion

The culture of bullying in nursing is prevalent internationally. Nursing students are vulnerable and often experience or observe bullying, leading them to question their future in nursing's “caring” profession. This study aimed to investigate the frequency of bullying and its relationship to the emotional state of 286 undergraduate nursing students in selected universities in the western region of Saudi Arabia.

The study revealed that most undergraduate nursing students (90.1%) reported exposure to bullying over the past 12 months; however, they had a relatively low frequency of exposure (*M* = 22.9, SD = 19.6 out of a total of 120). Reflecting these findings, a previous study in Saudi Arabia reported that most female students experienced bullying in their nursing education [24]. Existing literature indicates a large discrepancy in bullying prevalence. A recent literature review of 30 articles on bullying among nursing students reported that the prevalence varied from 9 to 96% [31]. In a Turkish study, 60% of nursing students reported that they had experienced at least one of 13 bullying behaviors at daily and weekly frequencies during the last six months [32]. A study conducted in eastern Saudi Arabia reported an almost 50% rate of exposure to bullying among medical and nonmedical university students [6]. International studies have reported a 50.1% prevalence of bullying among Australian undergraduate nursing students and a 35.5% prevalence among UK students [33]. In a Canadian study, Clarke et al. [28] reported that 88.7% of nursing students had experienced at least one act of bullying. The prevalence of bullying in these studies appears to be less than in this study. This may be due to the ratio of males to females in medical, nonmedical, and nursing studies. Most of this study's participants were female; generally, females experience more bullying than males [6]. The difference in bullying prevalence may also be due to the variation of questionnaires used in each study, which may only examine exposure to bullying in clinical settings.

This study found a high prevalence of depression, anxiety, and stress symptoms among Saudi nursing students. Most of the participants reported mild to extremely severe symptoms of depression (58.7%), anxiety (58%), and stress (44.8%). It is challenging to directly compare depression, anxiety, and stress levels in this study's sample with nonuniversity Saudi populations due to a lack of published studies in this area. Furthermore, any recent comparison of depression, anxiety, and stress levels in students must consider the psychological toll of the COVID-19 pandemic. This is relevant for this study as the data collection period was around 2020. For example, a study examining the prevalence of depression, anxiety, and stress among the general Saudi population revealed that the prevalence was 35.6%, 20.4%, and 23.0%, respectively, among people under 35 during the Covid-19 pandemic [34]. Another recent Saudi study using the DASS-21 tool revealed that nursing students showed lower rates of depression (43.3%), anxiety (37.2%), and stress (30.9%) compared to this study [35]. A recent Saudi study revealed relatively similar levels of depression, anxiety, and stress among medical students (55.8%, 45.8%, and 37.6%) and nonmedical students (52.1%, 60.2%, and 38.3%) compared to this study results [36]. An Australian study on nursing students in a public university in Sri Lanka identified a high prevalence of depression, anxiety, and stress symptoms. Most of the respondents reported mild to extremely severe symptoms of depression (51.1%), anxiety (59.8%), and stress (82.6) [37].

Regarding marital status, in accordance with our study, Sravani et al. [38] reported that married students showed significantly higher score of DAS than unmarried among undergraduate dental students. Interestingly in contrast to that, several studies conducted in Saudi Arabia found no significant relationship between undergraduates' marital status and their DAS [7, 22, 39]. More research is needed to clarify the effect of marital status on undergraduate students' emotions status in Saudi Arabia.

DASS-21 is a symptom-based scale in which the participants may exaggerate or underestimate their symptoms. This might lead to biased responses to these questionnaires, which may justify the differences between studies. Therefore, future research should be conducted to detect the prevalence of depression or anxiety disorders among nursing students. A recent study that used a different tool revealed that compared to their Australian and South African counterparts, Saudi Arabian nursing students suffered from more anxiety and depression and scored lower on the Mental Health Inventory [40]. This alarming finding highlights the increased risk of psychiatric morbidity among Saudi Arabian nursing students.

Nursing students face stressful events during their education that could negatively impact their psychological well-being. This is corroborated by Rathnayake and Ekanayaka [37], who stated that nursing students' stress is associated with a lack of professional knowledge and skills and lower clinical performance. Similarly, Shamsuddin et al. [41] stated that high academic expectations are stressful and can harm students' physical and mental health.

The findings of this study highlight the need for depression, anxiety, and stress management interventions and increased counseling facilities for nursing students. Furthermore, universities in Saudi Arabia should consider developing and implementing strategies to promote the emotional well-being of nursing students. As nurses constitute a large proportion of the health professional workforce, it is crucial to preserve their emotional well-being and prevent the long-term effects of negative emotions on nursing students.

Unexpectedly, the findings of this study revealed that third-year nursing students were more depressed, stressed, and anxious than other students. This contradicts the findings of Aboelyzeed [42] and Timmins et al. [43], who found that nursing students' stress and anxiety increased over the nursing program and were highest in the final year of study. More than half of their study subjects in their fourth and final years had the highest level of anxiety. They explained this finding by suggesting that increased stress in the last year of nursing education was responsible for elevating students' anxiety. This study's results are also contradicted by Wedgeworth [44], who studied the difference between prenursing, early nursing, and late nursing students and revealed that late nursing students had the lowest state and trait anxiety levels compared to prenursing and early nursing students. The prenursing and early nursing sample groups had the highest state and trait anxiety levels. Additional studies are needed on this topic to determine stress levels during academic semesters. Furthermore, nursing colleges should consider balancing the curriculum load across semesters.

The bullying experiences of nursing students were assessed using the 30-item bullying questionnaire. The most common items reported as part of bullying behavior included students' experiences of continually being assigned tasks beyond their capacity. This was followed by receiving unfair evaluations of their work and others underestimating the value of their academic work. Mohamed [24] stated that the most frequent bullying behaviors reported by Saudi female nursing students were verbal. These students were often threatened with poor evaluations and had their work belittled. Their significant clinical or academic achievements often went unacknowledged. AlMulhim et al. [6] noted a similar phenomenon among medical students. They found that the most common forms of bullying among this group were verbal abuse and unnecessary pressure to produce work.

In this study, the least common bullying behaviors reported by nursing students were being physically assaulted and unfairly treated because of race, incapacities, or weaknesses. Similarly, Mohamed [24] found that the least reported bullying behaviors were being treated poorly based on disability, racially motivated bullying, and physical abuse.

Unexpectedly, most students indicated that family members were the most significant source of bullying, followed by nursing faculty members (20.9%). In Mohamed's [24] study, around half of the respondents reported that a classmate was the most likely source of bullying, followed by faculty members and clinical instructors. After this, the most likely source of bullying was patients or patients' families. In addition, previous studies have reported that nursing students experienced and witnessed bullying behaviors at various frequencies, most notably by clinical instructors and staff nurses [28]. The discrepancy between this study and previous research could be due to the nature of the question that students were asked. In this study, they were asked an open-ended question, “what is the source of the bullying?” This allowed participants to discuss factors unrelated to nursing. Previous studies have restricted answers to this question by asking students to choose from specific categories, such as clinical settings and educational settings.

The link between family members and bullying suggests that interventions must start at home. Health professionals should ask about family bullying and intervene to prevent and reduce the health burden associated with this form of bullying. Furthermore, future research should examine the relationship between nursing students and nursing faculty members. Several initiatives have been introduced at Saudi Arabian universities to prevent this bullying. However, this study revealed that most nursing students did not read the list of students' rights or were unsure of its content.

Bullying victimization has recently emerged as a serious health concern for students. Bullying is often expected among students; however, its prevalence and effects may vary between ages or countries. Furthermore, although several studies have considered it a significant cause of stress and physical and emotional problems, its exact relevance to health and well-being is uncertain. This study showed that bullying behavior was positively correlated with students' scores for depression, anxiety, and stress. Bullying behavior was positively correlated with students' scores for depression, anxiety, and stress. Bullying behavior also predicted students' depression, anxiety, and stress. In accordance with our study, bullying behavior predicts the onset of emotional problems in adolescents [45], depressive symptomatology and suicidal ideation [46], anxiety [47, 48] and stress [49].

Setiadi et al. [50] analyzed the relationship between bullying and depression among undergraduate health students and found an association between bullying and the incidence of depression. Another study by Martínez-Monteagudo et al. [51] reported that being a cyberbullying victim increases the probability of suicidal thinking and leads to high levels of depression, anxiety, and stress. A previous study by Kowalski and Limber [52] indicated that depression, anxiety, self-esteem, and self-reported health problems are significantly related to bullying. Jadambaa et al. [53] stated that there is convincing evidence for a causal relationship between bullying victimization and mental disorders. They also found that bullying victimization significantly contributes to anxiety and depressive disorders. The results of a previous study indicated that university learning causes high anxiety levels in former and current victims of peer bullying. Unlike students who had never experienced bullying, victims reported more frequent anxiety and higher levels of context-specific social anxiety across various university learning environments.

A history of victimization and poor social relationships predicts the onset of emotional problems in adolescents [45]. A Ghanaian study found that bullying decreased confidence and caused stress and anxiety in nursing students [54]. Investing and implementing evidence-based intervention programs to reduce bullying victimization in Saudi Arabian nursing schools could reduce negative psychological symptoms and improve the mental health of Saudi Arabian nursing students.

### 4.1. Relevance for Clinical Practice

An in-depth understanding of different sources of bullying and their impact on emotional state allows clinical practitioners to reduce depression, anxiety, and stress in their respective areas. This information benefits all allied health practitioners by enabling administrators to develop awareness programs, preventive policies and procedures, training, and supportive measures. These measures can help to reduce emotional stress, which can lead to lower turnover rates and healthcare costs.

### 4.2. Recommendations

Nursing educators, academic advisors, and families should be educated to identify possible emotional signs of bullying, such as depression, anxiety, and stress. These groups should also increase their awareness of students' bullying behavior. Nursing schools need to educate students about the problem of bullying, and how to identify and respond to it, to prevent bullying from happening in the first place. Further research should include other universities in Saudi Arabia to collect relevant information and data on similar situations. This will further increase the understanding of bullying behavior and its impact on the emotional state of nursing students.

## 5. Conclusion

This study found a high prevalence of depression, anxiety, and stress symptoms among Saudi Arabian nursing students. Most of the respondents reported mild to extremely severe symptoms of depression (58.7%), anxiety (58%), and stress (44.8%). Around 90.1% of the nursing students reported exposure to bullying over the past 12 months with a relatively low frequency of exposure (*M* = 22.9, SD = 19.6 out of 120). The most common items reported as part of bullying were students' experiences of continually being assigned tasks beyond their capacity, followed by unfair evaluations of their work and other people underestimating the value of their academic work. Unexpectedly, most students indicated that family members were the most significant source of bullying, followed by nursing faculty members (20.9%).

## Figures and Tables

**Table 1:** Demographic characteristics of the sample.

Measure	*N*	*M*	SD
Age (years)	286	21	2.07
Measure	*N*	%	
Gender			
Male	50	17.5	
Female	236	82.5	
Marital status			
Single	274	95.8	
Married	10	3.5	
Divorced	2	0.7	
Nationality			
Saudi	283	99.0	
Non-Saudi	3	1.0	
Students' academic year			
First year	35	12.2	
Second year	34	11.9	
Third year	94	32.9	
Fourth year	123	43.0	
Have you read student rights manual?			
Yes	84	29.4	
No	133	46.5	
Unsure	69	24.1	
Bullying behaviors have negatively affected my appreciation of the nursing profession			
Yes	55	19.2	
No	163	57.0	
Not sure	68	23.8	

**Table 2:** Distribution of items on the bullying questionnaire (*N* = 286).

Items	Never		Other response		Mean score (0–4)	
	*n*	%	*n*	%	*M*	SD
(1) Angry yelling or shouting	100	35.0	186	65.0	1.08	0.99
(2) Using offensive language or inappropriate nonverbal signs in front of others	154	53.9	132	46.1	0.67	0.84
(3) Using teasing against you	111	38.8	175	61.2	1.09	1.07
(4) Using inappropriate nonverbal signals towards you and others	165	57.7	121	42.3	0.58	0.78
(5) Using belittling or undermining behavior for your work or efforts	110	38.5	176	61.5	1.22	1.17
(6) Underestimating the value of your academic efforts or work	110	38.5	176	61.5	1.22	1.18
(7) Spreading malicious rumours or allegations against you	206	72.0	80	28.0	0.44	0.85
(8) Threatening to give you a poor rating	168	58.7	118	41.3	0.66	0.93
(9) Denial of your academic achievement	165	57.7	121	42.3	0.83	1.20
(10) Threat of disciplinary action against you	199	69.6	87	30.4	0.50	0.90
(11) Unfair evaluation of your work or effort	88	30.8	198	69.2	1.40	1.20
(12) Excessive monitoring or constant criticism of your work	136	47.5	150	52.5	0.91	1.10
(13) Ironically making inappropriate jokes against you	178	62.2	108	37.7	0.68	1.02
(14) Assigning you tasks beyond your capacity continuously	105	36.7	181	63.3	1.44	1.40
(15) Setting expectations or impossible requirements for you	141	49.3	145	50.7	0.92	1.14
(16) Feeling ignored, marginalized, or physically isolated	175	61.2	111	38.8	0.67	1.00
(17) Changing your duties or tasks without being told	191	66.8	95	33.2	0.55	0.93
(18) Removing you from responsibilities without prior notice	251	75.2	71	24.8	0.35	0.71
(19) I was physically assaulted	259	90.6	27	9.4	0.11	0.39
(20) Deliberately humiliating you in front of others	194	67.8	92	32.2	0.46	0.79
(21) Putting you under undue pressure to produce	119	41.6	167	58.4	1.21	1.31
(22) Limiting your self-expression	162	56.6	124	43.4	0.76	1.09
(23) Trying to demoralize you	125	43.7	161	56.3	1.07	1.20
(24) Repeatedly reminding you of your mistakes	143	50.0	143	50.0	0.91	1.12
(25) Constant disregard for your opinions and points of view	138	48.3	148	51.7	0.88	1.10
(26) Hostile behavior	191	66.8	95	33.2	0.47	0.80
(27) Denial of learning opportunities	193	67.5	90	32.5	0.50	0.85
(28) I was treated badly or unfairly because of my race	245	85.7	41	14.3	0.23	0.67
(29) I was treated badly or unfairly because of my gender (i.e., being male or female)	195	68.2	91	31.8	0.64	1.09
(30) I have been treated badly or unfairly because of my incapacity or weakness	221	77.3	65	22.7	0.34	0.72

**Table 3:** Distribution of depression, anxiety, and stress scores (*N* = 286).

Variables	*M*						SD					
DASS total (21 items, Cronbach's *α* = 0.95)	20.9						15.7					

DASS subscales	Total score		Normal		Mild		Moderate		Severe		Extremely severe	
	*M*	SD	*n*	%	*n*	%	*n*	%	*n*	%	*n*	%

Depression (7 items, Cronbach's *α* = 0.90)	7.34	5.91	118	41.3	32	11.2	53	18.5	29	10.1	54	18.9
Anxiety (7 items, Cronbach's *α* = 0.86)	5.94	5.19	120	42.0	41	14.3	30	10.5	27	9.4	68	23.8
Stress (7 items, Cronbach's *α* = 0.90)	7.62	5.67	158	55.2	29	10.1	32	11.2	43	15.0	24	8.4

**Table 4:** Pearson correlation coefficient (*N* = 286).

Measures	1	2	3	4	5
(1) Bullying behavior	—				
(2) DASS total score	0.573^*∗∗*^	—			
(3) DASS depression	0.499^*∗∗*^	0.941^*∗∗*^	—		
(4) DASS anxiety	0.543^*∗∗*^	0.906^*∗∗*^	0.768^*∗∗*^	—	
(5) DASS stress	0.572^*∗∗*^	0.961^*∗∗*^	0.871^*∗∗*^	0.823^*∗∗*^	—
*M*	22.9	20.91	7.34	5.94	7.62
SD	19.6	15.7	5.91	5.19	5.67

**Table 5:** Analysis of variance of nursing students' academic year and the dependent variables (*N* = 286).

Measures	Nursing students' academic year								*F* (3, 282)	*p*
	First year (*n* = 35)		Second year (*n* = 34)		Third year (*n* = 94)		Fourth year (*n* = 123)			
	*M*	SD	*M*	SD	*M*	SD	*M*	SD		
(1) Bullying behavior	16.6	19.2	18.8	22.9	27.57	19.6	22.2	19.6	3.58	0.014
(2) DASS total	18.8	17.2	21.8	20.6	25.4	14.5	17.7	13.7	4.71	0.003
(3) DASS depression	6.97	6.74	7.97	7.30	9.06	5.60	5.96	5.12	5.27	0.001
(4) DASS anxiety	5.54	5.51	6.32	6.67	7.12	5.01	5.05	4.63	3.01	0.031
(5) DASS stress	6.28	5.74	7.55	7.22	9.28	5.21	6.74	5.26	4.47	0.004

## Data Availability

The data that support the findings of this study are available upon request.
